# Vineyard pruning-wood waste valorisation: sustainable extraction of bioactive compounds

**DOI:** 10.3389/fchem.2025.1597833

**Published:** 2025-06-05

**Authors:** Elisabetta Tumminelli, Valeria Cavalloro, Chiara Ingrà, Alessandra Ferrandino, Alessio Porta, Giorgio Marrubini, Emanuela Martino, Daniela Rossi, Simona Collina

**Affiliations:** ^1^ Department of Drug Sciences, University of Pavia, Pavia, Italy; ^2^ Department of Earth and Environmental Sciences, University of Pavia, Pavia, Italy; ^3^ National Biodiversity Future Center, Palermo, Italy; ^4^ DISAFA, Dipartimento Scienze Agrarie, Forestali, Alimentari, Università di Torino, Torino, Italy; ^5^ Department of Chemistry, University of Pavia, Pavia, Italy

**Keywords:** (*E*)-resveratrol, (*E*)-ε-viniferin enantiomers, UHPLC-UV/DAD analysis, microwave-assisted solvent extraction (MASE), secondary metabolites isolation, environmental impact factor (EF), AGREE: analytical GREennEss calculator

## Abstract

**Introduction:**

The annual production of waste is expected to increase over the next forty years, representing one of the main challenges associated with the global rise in population. Consequently, the transition towards more sustainable development and circular economy constitutes one of the most pressing challenges in the coming decades. Vineyard management generates several thousand tons of waste each year, including wood from pruning. This waste material is particularly rich in secondary metabolites, such as *(E)*-resveratrol and *(E)*-ε-viniferin. Accordingly, it represents a valuable source of biologically active phytochemicals with potential industrial outcomes.

**Methods:**

The present study aimed to exploit grapevine pruning residues as a source of *(E)*-resveratrol and *(E)*-ε-viniferin through the set-up of a low environmental impact procedure which involves first a microwave-assisted solvent extraction (MASE) followed by a protocol suitable for the isolation of *(E)*-resveratrol and *(E)*-ε-viniferin from the MASE extract. Different purification techniques, such as liquid/liquid extraction and chromatography, alone or in combinations, were exploited.

**Results and Discussion:**

Our optimized MASE protocol involves 100% EtOH as extraction solvent, 1 microwave cycle of 5 minutes at 80°C. As regards the isolation procedure, best results were achieved with medium pressure automated chromatography, eluting with n-hexane and ethyl acetate in gradient condition, with or without preliminary liquid/liquid (water/ethyl acetate) extraction. Applying the optimize procedure *(E)*-resveratrol (0.9 mg/g dry matrix weight) and *(E)*-ε-viniferin (1.1 mg/g dry matrix weight) were successfully isolated with high purity Moreover, a UHPLC-UV/DAD method suitable for the quantification of *(E)*-resveratrol and *(E)*-ε-viniferin was developed to support all the procedures. Keeping in mind eco-sustainable criteria, the greenness of the UHPLC method was evaluated through the open source calculator AGREE: analytical GREennEss Calculator 0.5 beta, while the environmental impact of the whole procedure proposed for the extraction and the isolation of the secondary metabolites was determined using the environmental impact factor (EF), obtaining satisfactory results.

## Graphical Abstract

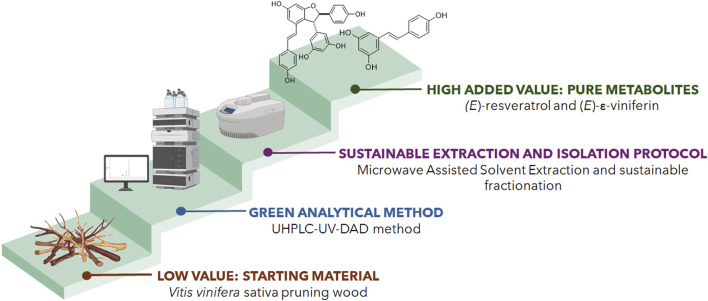

## 1 Introduction

According to the United Nations, annual waste production is set to increase by 70 percent over the next 40 years, thus representing a major concern related to the world’s growing population. Looking at Europe, cultivated land, grasslands, and pastures together account for about 39% of the soils used, thus being the main contributors to direct and indirect changes in the environment (European Environment Agency, 2025). Therefore, the transition to a more sustainable development and a sustainable economy is one of the most important challenges in the coming years (Lin et al., 2013).

In this scenario, the use of agro-industrial waste as a source of materials and natural substances with potential commercial interest could be a valuable perspective. So far, agricultural and food industry wastes have been described as important source of materials that find application in different sectors: production of biofilms (Li et al., 2022), biopolymers or biolipids (Sivakumar et al., 2022) or biochar (Kochanek et al., 2022), and extraction of bioactive molecule, to cite a few. Specifically, plant-based waste materials used for the extraction of bioactive molecules are well-detailed in the scientific and technical literature. To cite a few examples: *Sophora flavescens* Aiton, is a source of flavonoids and alkaloids that show anti-inflammatory properties *in vitro* (Chiocchio et al., 2021). *Prunus dulcis* (Mill.) D.A. Webb produces wastes containing polyphenols and flavonoids with antibacterial and antioxidant properties (Smeriglio et al., 2016). *Pinus pinaster* Aiton, whose principal agricultural waste product is bark, is a source of tannins that can be used as organocatalyst or tanning (Chiocchio et al., 2021). Within this context, wastes derived from *Vitis vinifera* L. cultivation must be cited, being rich in secondary metabolites of pharmaceutical interest belonging to different chemical classes (Sharafan et al., 2025).

*Vitis vinifera* L. *sativa* is widely cultivated worldwide, including Northern Italy, an area strongly devoted to agriculture and historically renowned for viticulture. Besides defining the wine production and quality, this area has a strong economic impact and has the capacity to bring further income from accessorizing activities. Examples are those based on the shaping of the landscapes, the realization of the wine districts, the communication of the vine-wine culture, and tourism. However, modern viticulture is associated with a strong environmental impact, mainly due to the vineyard management choices, the seasonal pathogen pressure, and the production of several thousand tons of waste each year, namely winery by-products and pruning wood (Zwingelstein et al., 2020). *Vitis* species accumulate in berries and in vegetative organs high amounts of flavonoid and non-flavonoid polyphenols. Those accumulated in berries have long time widely studied as they define the quality of fresh grapes and of future wines, conferring colour, astringency, bitterness, and enhancing or flattening specific gustative, olfactory, and visual traits of grapes and wines. Several researchers have studied the compositional traits and the possible application of winery by-products such as grape marc and seeds, as they still contain important amounts of anthocyanins, flavonols, flavan 3-ols, cinnamic and benzoic acids and their derivatives, stilbenoids (Goufo et al., 2020; Giampietro et al., 2014). Particularly, the grape marc is a rich source of several biologically active compounds responsible for the antioxidant properties of fermented grape marc (Živković et al., 2024).

**FIGURE 1:**
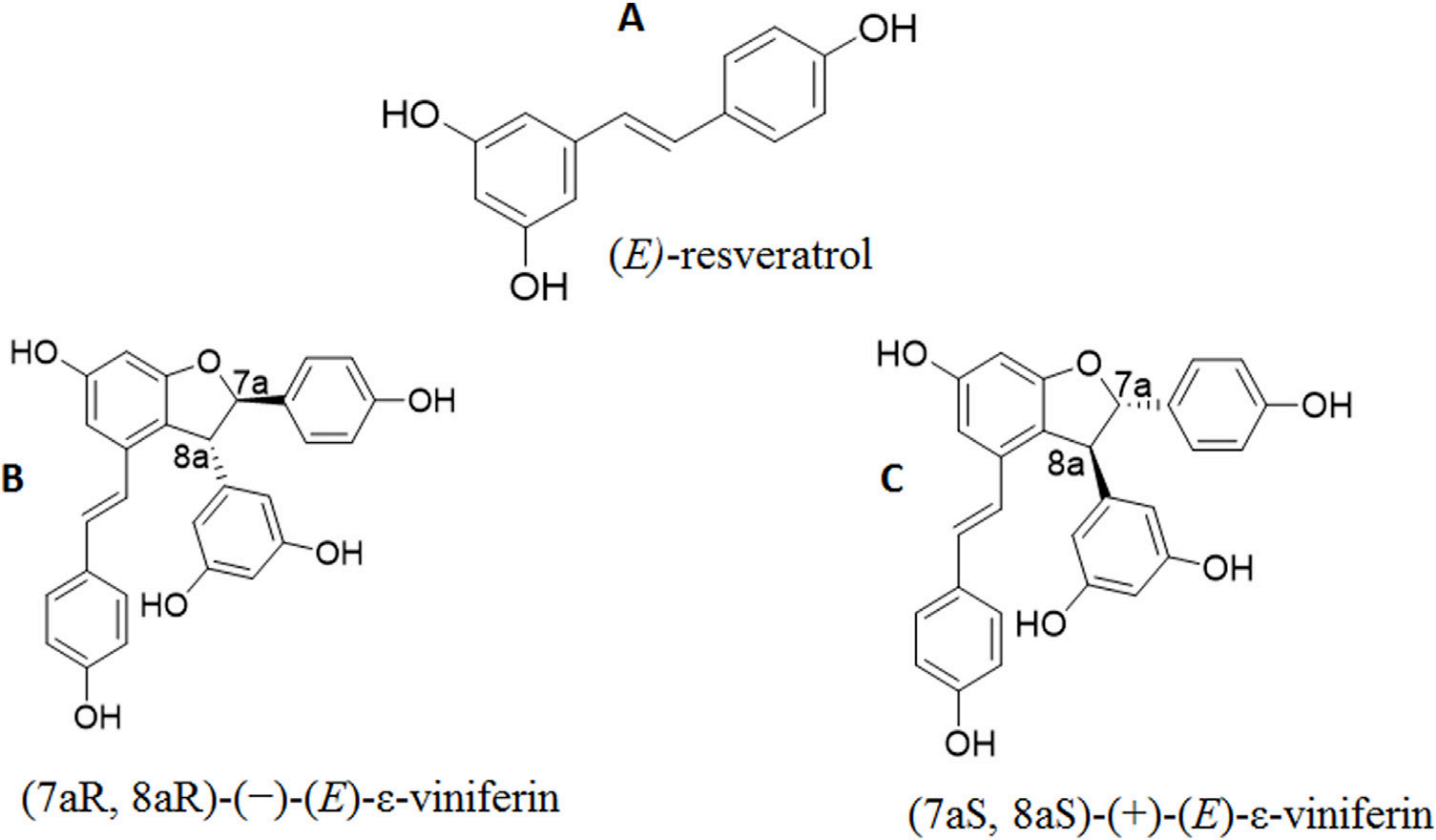
Structure of (*E*)-Resveratrol **(A)** and enantiomers of (*E*)-ε-viniferin **(B,C)**.

Much less is known about the pruning wood composition and possible exploitation. In the past years, notwithstanding the large amount of pruning wood produced in a vineyard, estimated in 1–5 tons/ha/year (Houillé et al., 2015), this resource was largely underestimated as commonly it was burnt in the vineyard (when it was still authorized) or chopped and buried. Up to now, viticultural pruning wood was proposed for the production of toasted chips to be used as additives in winemaking (Cebrián-Tarancón et al., 2018), for composting (Gaiotti et al., 2017) and for the extraction of bioactive molecules to be re-used in the winery process (Raposo et al., 2016) or to be destined to pharmaceutical/nutraceutical purposes (Donno et al., 2021). Stilbenoids are the most abundant polyphenols present in non-treated wood of *Vitis vinifera*; ecologically, their main role is defence against pathogens (fungi, bacteria) and, to a lesser extent, UV radiation. From a pharmacological standpoint (*E*)-resveratrol (Figure 1) displays antioxidant and anti-inflammatory (Meng et al., 2021) properties and has been proposed as a food supplement for preventing neurodegeneration and cardiovascular diseases (Bonnefont-Rousselot, 2016), and for improving intestinal barrier function (Sandoval-Ramírez et al., 2021) (*E*)-ε-viniferin is a stilbene dimer, frequently the most abundant polyphenol in non-treated canes of *Vitis vinifera* varieties (Houillé et al., 2015; Billet et al., 2025), characterised by the presence of two chiral centers (Figure 1). In the plant, it is mainly produced as a mixture of two enantiomers: (7a*R*, 8a*R*)*-*(*−*)-(*E*)-ε-viniferin (B, Figure 1) and (7a*S*, 8a*S)*-(+)-(*E*)-ε-viniferin (C, Figure 1), although in different proportions depending on the cultivar (Gabaston et al., 2023). Specifically, Cabernet sauvignon is rich in the (7a*S*, 8a*S*)-(+)-(*E*)-ε-viniferin (enantiomeric excess of 84.8%), while Syrah is rich in the (7aR, 8aR)-(−)-(*E*)-ε-viniferin (enantiomeric excess of 38.4%) (Gabaston et al., 2023). It is widely used as a cosmetic ingredient for its anti-staining and antioxidant action (Sáez et al., 2018). From a medicinal chemistry standpoint (*E*)-ε-viniferin enriched extracts were proposed for preventing obesity-related diseases, such as type 2 diabetes (Gómez-Zorita et al., 2023), and were studied for their anticancer potential due to anti-angiogenic and antioxidant actions (Tarhan et al., 2016). Although the enantiomers of a chiral molecule could behave differently in a biological environment, and thus present different biological activities, studies dealing with biological properties of (*E*)-ε-viniferin or (*E*)-ε-viniferin enriched extracts generally do not specify the enantiomeric composition of the secondary metabolite investigated. To the best of our knowledge, until now a very limited number of papers dealing with the enantiomeric composition of (*E*)-ε-viniferin in extracts obtained from different cultivars of *Vitis vinifera* have been published. Specifically, Gabaston and co-workers proposed the enantiomeric excess of (*E*)-ε-viniferin as a vine chemotaxonomic marker (Gabaston et al., 2023), and assessed the antioxidant and anti-inflammatory properties of its pure enantiomers (Buffeteau et al., 2022).

During the last years, conventional and nonconventional extraction methodologies, such as ultrasound-assisted or microwave-assisted extraction, have been exploited to prepare extracts rich in (*E*)-resveratrol and (*E*)-ε-viniferin from grape canes (Kapcsándi et al., 2021; Zuin and Ramin, 2018). Nevertheless, most studies do not propose any purification and isolation processes of the secondary metabolites from the crude extracts. A similar scenario emerged from a patent perspective. Many patent applications focus on the preparation of extracts enriched in (*E*)-ε-viniferin with different applications, but only a limited number describe the isolation of the pure metabolite. An example is patent WO2024096280A1 (Choi et al., 2024), which isolates (*E*)-ε-viniferin from grapevine stems. However, the procedure described in this patent has several flows, like long extraction time (1 week), the use of hazardous solvents (as dichloromethane), and of non-recyclable stationary phases. These considerations make the development of a more sustainable procedure of pivotal importance for considering vine wastes a sustainable source of ingredients with high intrinsic value, such as (*E*)-ε-viniferin.

Starting from the above considerations, the main objective of this work is the exploitation of grapevine pruning wood as a source of the (*E*)-resveratrol and (*E*)-ε-viniferin, which have high economic value and thus industrial outcome. To this purpose, following a green chemistry approach, a Microwave Assisted Solvent Extraction (MASE) procedure was firstly set up and optimized applying a Design of Experiment approach and, in parallel, a fast and low solvent consuming UHPLC-UV/DAD chromatographic method suitable for quantifying (*E*)-resveratrol and (*E*)-ε-viniferin in pruning wood extracts was developed. As the last goal of the work, a simple methodology suitable for isolating (*E*)-resveratrol and (*E*)-ε-viniferin from pruning wood extract was proposed.

## 2 Materials and methods

### 2.1 Plant material

One-year-old lignified shoots (=canes) of *Vitis vinifera* cv Syrah, were collected from 16-year-old vines in the collection vineyard of the University of Turin (DISAFA vineyard; 45 30530 0 N, 7350320 0 E) located in Grugliasco (TO), Italy, on the 2nd of February 2023. The DISAFA vineyard, planted in 2008, has a density of 4,400 vines/ha and is situated 293 m above sea level in a flat area. Grapevines are vertical shoot positioned and trained to the Guyot pruning system. The soil composition is sandy; further details can be found in Catoni et al. (2012). Syrah is grafted onto SO_4_ (*Vitis berlandieri* × *Vitis riparia*). Integrated agricultural management practices are applied. The integrated protocol for plant protection applied, beside using prevention and monitoring tools for the biocontrol of pathogens and weeds and the use of appropriate and well-trained cultural practices for the vineyard management, during the 2022 season implied the use of a treatment against *Scaphoideus titanus*, in line with the compulsory treatments indicated by Piedmont Region. To this aim, a non-systemic pyrethroid insecticide (lambda-cyhalothrin) was distributed in mid-July. No herbicides were distributed as the weed control was performed mechanically, through regular mowing of the spontaneous grass. During the period between the beginning of May and the end of July, a mixture of commercial fungicides (such as fluopicolide with fosetyl-Al and dimethomorph, against downy mildew; penconazole, against powdery mildew; dithianon and potassium phosphonate, against a broad spectrum of fungi, including *Plasmopara viticola*, *Phomopsis viticola* and *Guignardia bidwellii;* organic sulphur with foliar action) was distributed to the canopy. No fertilizers were distributed during the 2022 season or the first months of 2023. After collection, plant material was cleaned, cut, and left to dry in a ventilated stove at 35°C until constant weight and then stored in a dark, dry place. Before extraction, the plant material was ground finely.

### 2.2 Chemicals and standards

HPLC-grade solvents (Acetonitrile and Methanol) were supplied by Merck-Sigma-Aldrich (Milan, Italy), while *n*-hexane and 2-Propanol were supplied by VWR (Milan, Italy), analytical grade ethanol and ethyl acetate were supplied by PanReac (Barcelona, Spain). Formic acid was purchased from Sigma-Aldrich (Milan, Italy) (*E*)-Resveratrol and (*E*)-ε-viniferin were purchased from PhytoLab GmbH & Co. KG (Germany). Silica gel from Sigma-Aldrich (Milan, Italy).

### 2.3 Apparatus

Plant material was ground on Blade-mill (A10 IKA-Werke GmbH & Co., Staufen, Germany).

Extraction method was set up on microwave mono-mode oven (Discover^®^ Lab-Mate instrument, CEM Corporate, Buckingham, UK) equipped with a power and temperature controller.

Extraction method scale up was evaluated on microwave multi-mode oven (MARSX system, CEM Corporate, Buckingham, UK)

Sample solvent removal was performed using Heidolph Laborota 4,000 instrument (Heidolph Instruments GmbH & Co., Schwabach, Germany) and Smart Evaporator C1 (Stepbio, Bologna, Italy).

Pure metabolites isolation was carried out on Büchi Pure Chromatography System C-810 system (BUCHI Italia s.r.l, Cornaredo IT.) including a quaternary pump, UV-Vis-wavelength range of 200–800 nm, software version 1.8.1000.29664.

^1^H NMR spectra were recorded on Bruker 400 spectrometer with ^1^H at 400.134 MHz and ^13^C at 100.62 MHz. Proton chemical shift was referenced to the residual solvent peak. Chemical shifts are reported in parts per million (ppm and δ units). Coupling constants are reported in units of Hertz (Hz). Splitting patterns are designated as follows: s, singlet; d, doublet; t, triplet; q quartet; dd, double doublet; m, multiplet; b, broad.

Analytical thin layer chromatography (TLC) analyses were carried out on silica gel pre-coated glass-backed plates (TLC Silica Gel 60 F254, Merk) impregnated with a fluorescent indicator, and visualized with the instrument MinUVIS, DESAGA^®^ Sastedt-GRUPPE by ultraviolet (UV) radiation from UV lamp (λ = 254 and 366 nm)

#### 2.3.1 Chromatographic systems

System A. Jasco UHPLC-UV/DAD system (Jasco Europe, Cremella, Italy) equipped with a quaternary pump model PU-4180, autosampler model AS-4050 with a 5 μL loop, column thermostatic compartment model CO-4065; UV-Vis photodiode array detector (PAD) model MD-4010 with a semi-micro cell (Jasco 2023), ChromNAV software (version 2.04.03).

System B. UHPLC apparatus JASCO (Lecco, Italy) X-LC system coupled with a MS spectrometer LTQ XL HESI-MS/MS system. HESI determinations were performed with MS spectrometer Thermo Scientific (Milan, Italy) LTQ XL HESI-MS/MS system using UHPLC-Flow injection of purified fractions. HESI Probe: Gas = N_2_, T = 95°C, Voltage = 3.5kV; Capillary T = 275°C, Voltage = 46V, Tube Lens = 71V. Tune Settings: Multipole 00 Offset = 2.5V, Lens 0 = - 4.27V, Multipole 0 Offset = −5.19 V, Lens 1 = - 8.93, Gate Lens = - 65.3 V, Multipole 1 Offset = - 6.4 V, Multipole RF Amplitude (p-p) = 400 V, Front Lens = - 6.2 V. Settings for MS-MS and MS^3^: detection by CID (Collision Induced Dissociation); Isolation Width: ±2 days; Activation Q: 0.250; Activation Time 30.0 msec. ChromNAV software (version 2.03.02) and Xcalibur (version 4.2).

System C. Jasco HPLC system (Jasco Europe, Cremella, Italy) equipped with a quaternary pump model PU 2089 Plus with autosampler model AS 2055 Plus associated 100 μL loop, UV-Vis photodiode array detector (DAD) model MD 1510, Electronic Circular Dichroism (ECD) detector model CD-2095 plus and ChromNAV software (version 2.04.03).

### 2.4 MASE parameters determination through design of experiments (DoE) approach

In the experimental extraction plan three factors were evaluated: solvent composition *(X*_*1*_ *)* water/ethanol from 20/80 (−1) to 80/20 (+1), duration of the MASE cycle (*X*_*2*_), from 5 (−1) to 15 (+1) minutes, and temperature *(X*_*3*_ *),* from 40°C (−1) to 80°C (+1). Given these factors and levels, a full factorial design including eight experiments was used as an experimental plan (Table 1). Three additional experiments in the central point (*X*_1_ = *X*_2_ = *X*_3_ = 0) were performed to validate the model. All the other parameters were kept constant, including drug/solvent ratio of 20% (400 mg of the matrix extracted with 2 mL of solvent), MW power of 50 Watts, maximum pressure of 120 PSI, ramp time of 2 min, number of cycles equal to 1, and magnetic stirring. After each extraction, the mixture was allowed to cool to room temperature and filtered using vacuum filtration with a Buchner funnel. To wash the filter, 4 mL of solvent was applied, varying in composition according to the experimental plan. All samples were dried until constant weight to determine the total extraction yield (*Y*_*3*_) and then analysed by UHPLC-UV/DAD (system A). Each extract was dissolved in an 80/20 (v/v) methanol/water mixture reaching a final concentration of 10 mg mL^−1^, analyzed by UHPLC and filtered through a 0.45 μm GH Polypro membrane (GHP-PerkinElmer, China) before injection into the UHPLC-UV/DAD system for the quantitation of (*E*)-resveratrol, and (*E*)-ε-viniferin through calibration curve (responses *Y*_1_ and *Y*_2_, respectively). The experimental plan and matrix are shown in Table 1. Following the DoE experiments and data analysis, one final experiment was carried out at fixed settings of cycle duration and temperature of 5 min and 80°C, respectively, to assess the effect of 100% ethanol on the responses *Y*_1_ and *Y*_2_.

**TABLE 1:** DoE experimental plan.

Exp#	*X*_*1*_: Composition of solvent (water/ethanol) ratio	*X*_*2*_: Time of extraction	*X*_*3*_: Temperature
1	−1	−1	−1
2	1	−1	−1
3	−1	1	−1
4	1	1	−1
5	−1	−1	1
6	1	−1	1
7	−1	1	1
8	1	1	1
9	0	0	0
10	0	0	0
11	0	0	0

### 2.5 Statistical analysis

All data were studied using Microsoft^®^ Excel^®^ for Microsoft 365 MSO (Version 18.2002.1101.0) and R for Microsoft Windows version 3.2.3, Copyright^©^ 2014. The R-based software CAT (Leardi et al., 2025) was used for DoE calculations.

### 2.6 Extraction scale up

To assess the scalability of the extraction method, the Microwave multi-mode oven was used. The extraction was performed in triplicate on 60 g of *Vitis vinifera* cv Syrah one-year-old pruning wood. The instrument set up was: power 320 W, maximum pressure of 120 PSI, ramp time of 2 min, hold time 5 min, 1 cycle, 80°C. The mixture was allowed to cool to room temperature and then filtered through vacuum filtration. Each extract was dissolved in an 80/20 (v/v) methanol/water mixture (10 mg mL^−1^) and filtered through a 0.45 μm GH Polypro membrane (GHP-PerkinElmer, China) before UHPLC-UV/DAD analysis (system A).

### 2.7 Metabolite isolation

Method A: Crude extract (2 g) was suspended in water (300 mL) and extracted with ethyl acetate (3 x 300 mL). The organic phases were dried with Na_2_SO_4_ and evaporated under reduced pressure and then fractionated by medium-pressure automated chromatography on a Büchi Pure Chromatography System C-810 system, using a silica cartridge (330g, 30 mL column volume, particle size 40–63 µm, pore size 55–75 Å) eluting with *n*-hexane (solvent A) and ethyl acetate (solvent B) in gradient mode. Gradient conditions: from 100% A to 50% A in 10 min, followed by an isocratic elution phase for 2 min, from 50% A to 40% of A in 2 min, followed by isocratic elution keeping this latter condition for 10 min. Fractions containing (*E*)-ε-viniferin and (*E*)-resveratrol (TLC analysis) were evaporated to dryness.

Method B: Crude extract (2 g) was suspended in water 300 mL and extracted with ethyl acetate (3 × 300 mL). The organic phases were dried with Na_2_SO_4_, evaporated under reduced pressure and subjected to flash chromatography on silica gel, employing a gradient elution starting with 50% of *n*-hexane (solvent A) and 50% of ethyl acetate (solvent B), followed by 40% A. Fractions containing (*E*)-ε-viniferin and (*E*)-resveratrol (TLC analysis) were evaporated to dryness.

Method C: Crude extract (2 g) was fractionated directly by medium-pressure automated chromatography on a Büchi Pure Chromatography System C-810 system, using a silica cartridge (330g, 30 mL column volume, particle size 40–63 µm, pore size 55–75 Å) and eluting with *n*-hexane (solvent A) and ethyl acetate (solvent B) in gradient mode. Gradient conditions: from 100% A to 50% A in 10 min, followed by an isocratic elution phase for 2 min, from 50% A to 40% of A in 2 min, followed by isocratic elution keeping this latter condition for 10 min. Fractions containing (*E*)-ε-viniferin and (*E*)-resveratrol (TLC analysis) were evaporated to dryness.

Method D: Crude extract (2 g) was fractionated directly by flash chromatography on silica gel employing a gradient elution starting with 50% of *n*-hexane (solvent A) and 50% of ethyl acetate (solvent B), followed by 40% A. Fractions containing (*E*)-ε-viniferin and (*E*)-Resveratrol (TLC analysis) were evaporated to dryness.

(*E*)-ε-viniferin: pale green powder; C_28_H_22_O_6_; mp 63°C; m/z 455.28; UHPLC-UV/DAD purity 98%; ee% = 60; the ^1^H-NMR data agree with published data (Buffeteau et al., 2022).

(*E*)-resveratrol: white powder; C_14_H_12_O_3_; mp 261°C; m/z 228.11; UHPLC-UV/DAD purity 99%; the ^1^H-NMR data agree with published data (Koh et al., 2001).

### 2.8 Chromatographic analysis

#### 2.8.1 Method development and validation

Chromatographic conditions were developed and optimized on system A, using the Purospher^®^ STAR RP-18 endcapped column (2.1 mm ID × 100 mm, particle size 3 μm) (Merck, Darmstadt, Germany). The mobile phase consisted of water containing 0.1% (v/v) formic acid (solvent A) and acetonitrile containing 0.1% (v/v) formic acid (solvent B). The elution was in gradient mode as follows: from 5% to 40% of solvent B in 4 min, from 40% to 50% of solvent B in 9 min, up to 80% of solvent B in 7 min, followed by an isocratic elution phase for 5 min. The column was reconditioned by eluting from 80% of solvent B to 5% of solvent B in 0.1 min, followed by a final 15 min isocratic elution at the initial conditions (5% of B). The analyses were performed at 25°C and at a flow rate of 0.2 mL min^−1^. The injection volume was 1 μL.

Linearity, detection limit (DL) and quantitation limit (QL) and repeatability of the UHPLC-UV/DAD method were evaluated according to the ICH Q2 (R2) (U.S. Food and Drug Administration, 2024; European Medicines Agency, 2024) guidelines.

Stock solutions of (*E*)-resveratrol (c = 1 mg mL^−1^) (*E*)-ε-viniferin (c = 0.83 mg mL^−1^) were prepared in 80/20 (v/v) methanol/water mixture, diluted to purpose and filtered through 0.45 μm PTFE membranes before analysis.

The calibration curves were built through ten points, each replicated three times, in the range of 800–0.10 μg mL^−1^ and of 726–0.10 μg mL^−1^ for (*E*)-resveratrol and (*E*)-ε-viniferin, respectively.

The limits of detection (DL) and quantification (QL) were estimated through specific calibration curves built within the range 0.1 μg mL^−1^ and 2.5 μg mL^−1^, from which the average slope (S) and the standard deviation of intercept were computed, according to ICH guideline Q2(R2) on validation of analytical procedures (U.S. Food and Drug Administration, 2024; European Medicines Agency, 2024). The following formulas were applied:

$$DL=\frac{3.3\sigma }{S}$$

$$QL=\frac{10\sigma }{S}$$

where 

σ = standard deviation of y-intercepts of the regression lines.

S = slope of the calibration curve.

The repeatability of the UHPLC-UV/DAD method was verified by performing 10 determinations at the test concentration of 250 μg mL^−1^ and 259 μg mL^−1^ for (*E*)-resveratrol and (*E*)-ε-viniferin, respectively.

#### 2.8.2 Samples analysis

Samples were prepared dissolving crude extract (c ∼10 mg mL^−1^) or the pure secondary metabolites (c ∼0.5 mg mL^−1^) in 80/20 (v/v) methanol/water mixture and filtered through 0.45 μm PTFE membranes before analysis on system A or system B.

#### 2.8.3 Enantioselective chromatography

Analytical chiral resolution of (*E*)-ε-viniferin was carried out on system C using a Chiralpak IA (4.6 mm Ø × 25 cm, 5 µm) column, eluting with *n-*hexane added with 0.01% (v/v) TFA (solvent A) and 2-Propanol, IPA (solvent B) added with 0.01% (v/v) TFA at a flow rate of 1 mL min^−1^ in isocratic mode at 20% B (0–23 min). Sample solutions were prepared by dissolving analytes at ∼ 0.5 mg mL^−1^ in 80/20 (v/v) n-hexane/IPA and filtered through 0.45 μm PTFE membranes before analysis. The injection volume was 1 µL. Chromatography analyses were performed at room temperature.

The enantiomeric excess (ee) was calculated as follows:

ee%=% enantiomer 1−% enantiomer 2

where

% enantiomer 1+% enantiomer 2=100%

## 3 Results

### 3.1 UHPLC-UV/DAD method development

**FIGURE 2:**
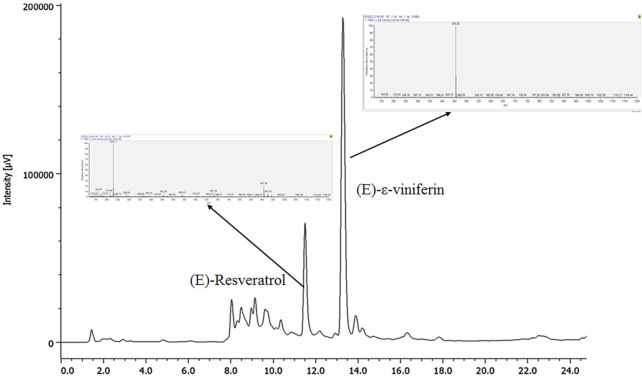
UV trace (λ: 306 nm) of the pilot extract, with the main secondary metabolites indicated together with their patterns of fragmentation.

The UHPLC-UV/DAD method development was performed using a pilot extract of *Vitis vinifera* cv. Syrah one-year-old pruning wood prepared for purpose according to Ingrà et al. (2024). With the aim to develop a UHPLC-UV/DAD procedure suitable for a quick quantitation of (*E*)-resveratrol and (*E*)-ε-viniferin in *Vitis vinifera* pruning wood extracts, several reverse columns as well as different elution conditions, both in isocratic and gradient modes, were considered in our preliminary experiments. Among the tested columns of equivalent format, Purospher STAR RP-18 endcapped column gave the best results, allowing to achieve a complete resolution of (*E*)-resveratrol and (*E*)-ε-viniferin from the other metabolite present in the extract in reasonable analysis times (24 min, Figure 2) and with a low solvent consumption. The column is made of high-purity type B silica that allows all-around retention features; it is characterized by a good range of pH stability (from pH 1.5–9.5) as well as a wide temperature range compatibility along with suitability for up to 100% aqueous mobile phases. Optimized elution conditions consisted of 0.1% (v/v) formic acid (A) and acetonitrile containing 0.1% (v/v) formic acid (B), in gradient mode as described in paragraph 2.8.1. Analysis evidenced that the extract contains two main secondary metabolites (*E*)-resveratrol (t_R_ = 11.4 min) and (*E*)-ε-viniferin (t_R_ = 13.3 min), as confirmed by UV spectra and MS patterns of fragmentation and retention times of authentic reference standards (Figure 2). The selectivity was ensured by the photodiode array detector and confirmed by UHPLC/MS analysis.

To properly quantify (*E*)-resveratrol and (*E*)-ε-viniferin present in pruning wood extracts, the UHPLC-UV/DAD method was validated in terms of linearity, detection limit (DL), quantitation limit (QL), and repeatability, according to the ICH Q2(R2) and USFDA guidelines on validation of analytical procedures. The method response function resulted to be linear for both (*E*)-resveratrol (y = 26060x + 157104; R^2^ = 0.9974) and (*E*)-ε-viniferin (y = 119,43x + 40138; R^2^ = 0.9992) within the concentration range investigated (800–0.10 μg m L^−1^ and of 726–0.10 μg m L^−1^ for (*E*)-resveratrol (*E*)-ε-viniferin, respectively). The QL was 0.193 μg m L^−1^ and 0.212 μg mL^−1^ for (E)-Resveratrol (E)-ε-viniferin, respectively (Supplementary Figure S1). The assess repeatability of the UHPLC-UV/DAD method, ten determinations at the test concentrations (250 μg mL^−1^ for (*E*)-resveratrol and 259 μg mL^−1^ for (*E*)-ε-viniferin) were carried out, furnishing %RSD of the area values of 0.9% for (*E*)-resveratrol and (*E*)-ε-viniferin, thus confirming that the method was reproducible.

### 3.2 Extraction and isolation of (*E*)-resveratrol and (*E*)-ε-viniferin

For the extraction of (*E*)-resveratrol and (*E*)-ε-viniferin from *Vitis vinifera* canes, a MASE protocol was developed through a DoE approach. All experiments were conducted using *Vitis vinifera* cv Syrah. The experimental plan was designed to study three responses, namely, the mg of (*E*)-resveratrol (*Y*_1_) and (*E*)-ε-viniferin (*Y*_2_) per g of dry matrix weight (DW) and the total extraction yield (*Y*_3_), calculated as mg of dry extract per mg of dried plant material (DW) × 100 (Table 2).

**TABLE 2:** DoE experimental plan and response data.

Exp#	*X*_*1*_: Solvent composition (water/ethanol) ratio	*X*_*2*_:time of extraction (min)	*X*_*3*_: Temperature (°C)	*Y*_*1*_: (E)-resveratrol (mg/g DW)^a^	*Y*_*2*_: (E)-ε-viniferin (mg/g DW)^a^	*Y*_*3*_: yield (mg/g DW)%
1	20/80	5	40	0.21	1.24	5.5
2	80/20	5	40	ND^b^	0.11	3.7
3	20/80	15	40	0.17	0.85	3.8
4	80/20	15	40	ND^b^	0.12	4.0
5	20/80	5	80	0.19	0.96	4.9
6	80/20	5	80	ND^b^	0.07	3.4
7	20/80	15	80	0.30	1.23	5.9
8	80/20	15	80	ND^b^	0.05	2.4
9	50/50	10	60	0.15	0.97	2.5
10	50/50	10	60	0.15	1.13	2.6
11	50/50	10	60	0.21	1.35	4.6

^a^ Metabolites were quantified by UHPLC-UV/DAD through calibration curves in the crude extract.

^b^ ND, value below a quantitation limit.

Preliminary analyses were performed to identify the most important variables to include in the experimental plan. In detail, the number of microwave cycles, drug/solvent ratio, extraction solvent composition, MW power, and temperature were evaluated (data not shown). In the end, only temperature, cycle duration, and extraction solvent composition showed impact on the final yields, and thus these factors were considered to set up the experimental model. In detail, as extraction solvent a mixture of water and ethanol was exploited, with a composition (*X*_*1*_) ranging from a minimum of 80% EtOH to a maximum of 80% H_2_O. The duration of the MASE cycle (*X*_*2*_ *)* ranged from 5 to 15 min, whereas the temperature (*X*_*3*_ *)* was exploited from 40°C to 80°C (Table 2). The levels of all these parameters have been selected based on both our preliminary experiments and data available in the literature. All other parameters, including the operator, were kept constant.

The multilinear regression models computed were validated by performing three test experiments in the domain centre (*X*_*1*_ = *X*_*2*_ = *X*_*3*_ = 0) to verify whether the models computed for the responses reliably represented the responses and assess the overall method standard deviation. The results demonstrated that the models were able to predict both *Y*_*1*_ and *Y*_2_ and that only the percentage of ethanol significantly influenced the responses. In detail, a higher ethanol percentage corresponds to higher responses *Y*_*1*_ and *Y*_*2*_. As far as cycle duration and temperature are concerned, within the ranges of variation studied here, they had no significant effect on *Y*_*1*_ or *Y*_*2*_. Finally, none of the three factors, *X*_*1*_, *X*_*2*_, and *X*_*3*_, significantly affected the total extraction yield (*Y*_*3*_), thus demonstrating that this response is robust under the experimental conditions applied within the experimental domain. Considering the conclusions reached after the DoE study, to assess the effect of the extraction solvent on *Y*_*1*_ and *Y*_*2*_, one final experiment was performed at 5 min and 80°C, using 100% ethanol. The results of this latest experiment suggested a trend of improvement in both the responses *Y*_*1*_ and *Y*_*2*_ (0.77 mg/g DW for (*E*)-resveratrol and 1.40 mg/g DW for (*E*)-ε-viniferin), which was confirmed by carrying out this experiment in triplicate. These optimized extraction conditions were then applied to 60 g of the pruning wood matrix using a multimodal mw-oven as described in the experimental section (paragraph 2.6), to evaluate the scalability of the MASE method. Results summarized in Table 3 clearly indicate that the change of scale and type of mw-oven did not affect the extraction efficiency of the method. With a view to large-scale application, recent advances in the microwave apparatus led to the development of a microwave extractor suitable for processing kilos of biomass. This, associated with the low extraction time of our method, would allow to process a high amount of biomass, thus overcoming scalability issues usually associated with MASE.

**TABLE 3:** Comparison between the two different extraction scales exploited (about 400 mg and about 60 g of pruning wood). Results are expressed as mean of three experiments ± SD.

Amount of matrix (pruning wood)	(E)-Resveratrol mg/g DW^a^	(E)-ε-Viniferin mg/g DW^a^
∼ 400 mg	0.77 ± 0.03	1.40 ± 0.06
∼ 60 g	0.81 ± 0.01	1.36 ± 0.02

^a^ Metabolites quantified by UHPLC-UV/DAD through calibration curves in the crude extract.

**FIGURE 3:**
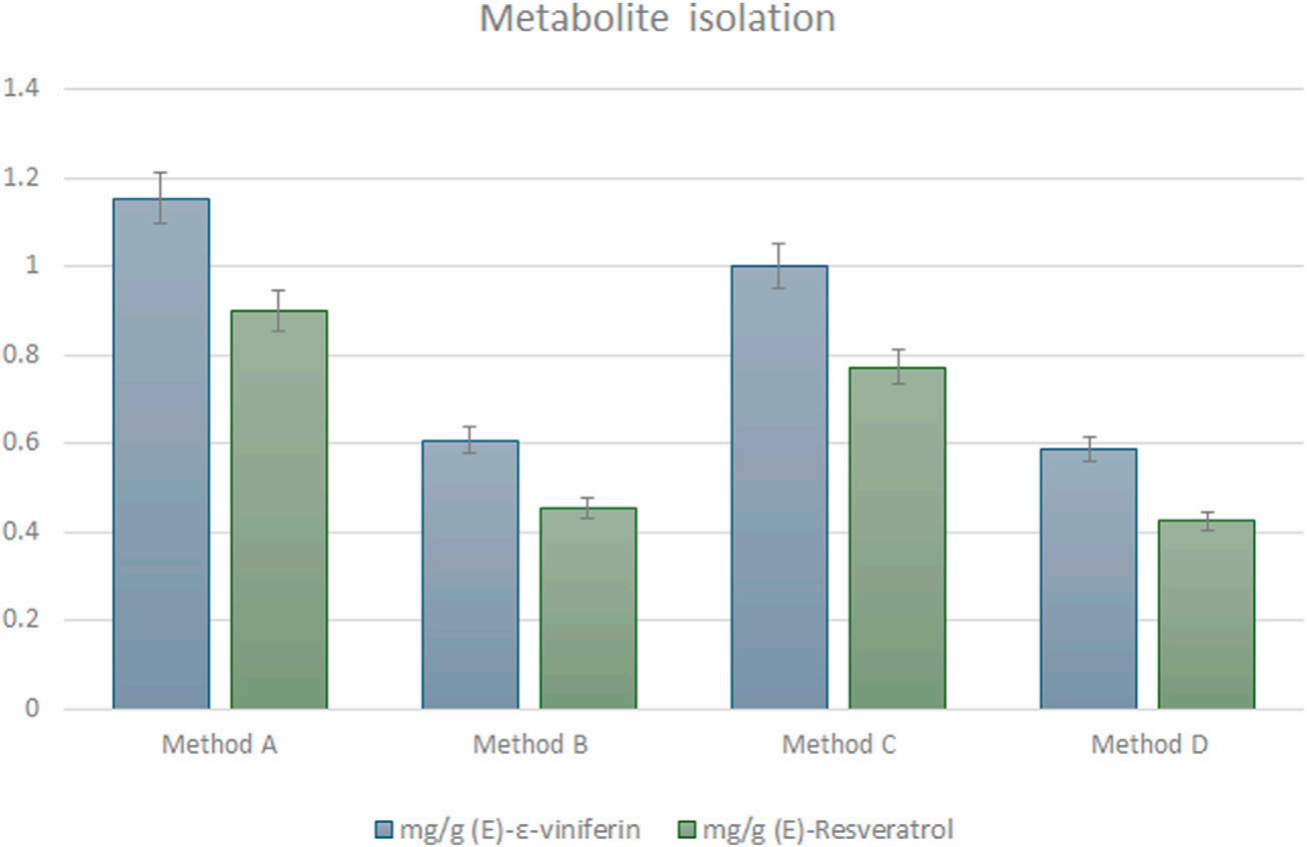
Comparison of methods A-D exploited for isolating (*E*)-resveratrol ad (*E*)-ε-viniferin from the MASE extract (paragraph 2.7). Results are expressed as mg/g of pure secondary metabolite (mean of three experiments ± SD) isolated from 40 g of dried pruning wood.

Finally, to isolate (*E*)-resveratrol and (*E*)-ε-viniferin from the MASE extract, four procedures were exploited (methods A-D, experimental section paragraph 2.7), which differ for both i) the presence or the absence of a preliminary liquid/liquid (L/L) (water/ethyl acetate) extraction step before the isolation through chromatographic methodology and ii) the type, medium pressure automated or flash manual, of the chromatography employed. Ethyl acetate was selected for the L/L extraction due to its effectiveness in obtaining fractions enriched in resveratrol and related compounds WO2012156917A2 (Vercauteren and Salmi, 2012). The best results were achieved with medium pressure automated chromatography, eluting with *n*-hexane (solvent A) and ethyl acetate (solvent B) in gradient conditions, with or without preliminary L/L extraction (method A and C respectively, paragraph 2.7) (*E*)-resveratrol elutes at 50% of solvent A, while(*E*)-ε-viniferin elutes at 40% of solvent A. Yields of methods A and C are ≈ 0.9 mg/g DW for (*E*)-resveratrol (36 mg and 31 mg of pure metabolite isolated from 40 g of dried matrix for method A and C, respectively) and ≈ 1.1 mg/g DW for (*E*)-ε-viniferin (46 mg and 40 mg of pure metabolite isolated from 40 g of dried matrix for method A and C, respectively). On the other hand, manual chromatography performed either directly on the crude extract or after L/L extraction (method B and D, respectively paragraph 2.7) significantly decreases the yields to ≈ 0.4 mg/g DW for (*E*)-resveratrol (18 mg and 17 mg of pure metabolite isolated from 40 g of dried matrix for method B and D, respectively) and to ≈ 0.6 mg/g DW for (*E*)-ε-viniferin (24 mg and 23 mg of pure metabolite isolated from 40 g of dried matrix for method B and D, respectively), probably due to the higher percentage of non-pure fractions obtained during the chromatographic separation (Figure 3). In all cases, both the stilbenoids were isolated with high chemical purity (>98%), as confirmed by UHPLC-UV-DAD analysis. Further considerations related to method greenness will be done in the discussion session.

**FIGURE 4:**
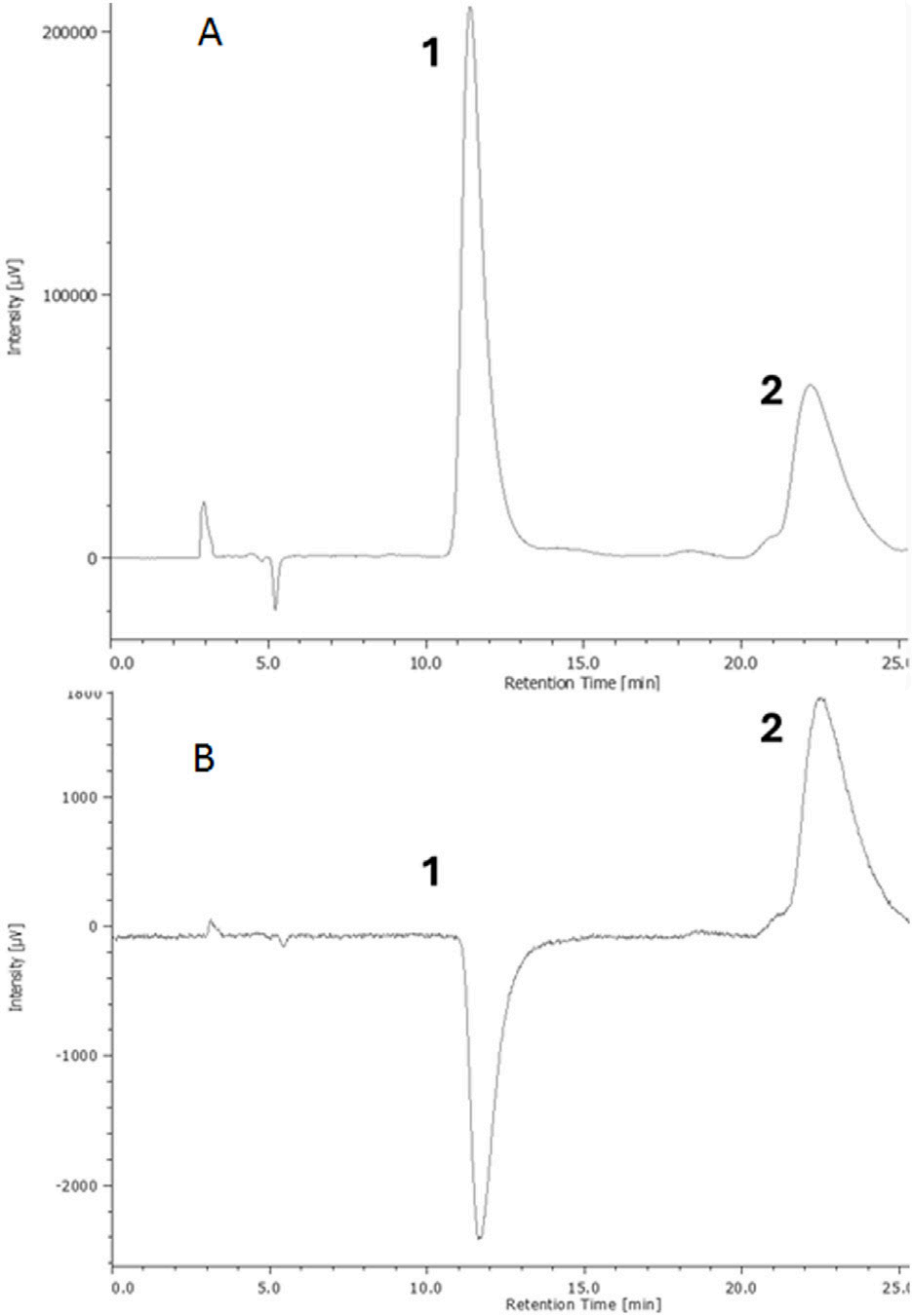
UV trace at λ 306 nm **(A)** and ECD trace at λ 240 nm **(B)** of (*E*)-ε-viniferin enantiomers from *Vitis vinifera* cv. Syrah. Chiral stationary phase: Chiralpak IA column; elution conditions: 80% n-hexane (solvent A) and 20% 2-propanol (solvent B) both added with 0.01% (v/v) TFA, flow rate 1 mL min^−1^.

To properly characterize the isolated metabolites, the enantiomeric excess of (E)-ε-viniferin was determined by analytical enantioselective chromatography couple with UV/DAD and ECD detectors. Briefly, baseline resolution was achieved using Chiralpak IA column, eluting with 80% of *n*-hexane and 20% of 2-propanol, both added with 0.01% (v/v) TFA, at a flow rate of 1 mL min^−1^ (Figure 4). The UV trace evidenced the presence of two peaks related to the two (*E*)-ε-viniferin enantiomers (1 and 2, respectively, Figure 4A), which showed opposite signals in the ECD trace (Figure 4B). The enantiomeric excess of the most abundant enantiomer 1 is 60%. Basing on the work of Buffeteau and co-workers, comparing both the elution order on chiral columns which are made by the same chiral selector [amylose tris (3.5-dimethylphenylcarbamate)] and the sign of the ECD signals, we assigned the absolute configuration (AC) to 1 and 2. Briefly, the AC of the enantiomers of (*E*)-ε-viniferin extracted from *Vitis vinifera* was previously assigned, after isolation on semi-preparative Phenomenex Lux Amylose-1, combining NMR spectroscopy, vibrational circular dichroism (VCD) and theoretical calculations approach (Buffeteau et al., 2022). The configuration (7aS, 8aS) was assigned to the second eluted enantiomer, which showed a positive ECD curve in the range of wavelength between 220 and 260. As reported above, in our experimental conditions (*E*)-ε-viniferin enantiomers were successfully resolved on Chiralpak IA column, which is made by the same chiral selector of the Phenomenex Lux Amylose-1, with the only difference that it is immobilized on silica, under the same elution conditions. In our experiments the first eluted enantiomer (1) showed a negative CD signal at 240 nm, while the second eluted one (2) showed a positive signal at the same wavelength (Figure 4B). Accordingly, based on both the elution order and ECD signals, the (7a*R*, 8a*R*) AC was assigned to 1 and the (7a*S*, 8a*S*) AC to 2.

## 4 Discussion

The demand for green and cost-effective protocols suitable for the reuse of agricultural waste with a reduction in both energy consumption and use of non-environmentally friendly solvents has increased in the last few years, according to the need for a transition to more sustainable development and a sustainable economy. In this scenario, we focused on the development of a combined microwave-assisted solvent extraction (MASE)- ultra high pressure liquid chromatography (UHPLC) approach suitable for a quick identification of the best experimental conditions to maximize the extraction of (*E*)-resveratrol and (*E*)-ε-viniferin from *Vitis vinifera* one-year-old pruning wood, one of the most abundant waste in viticulture. Moreover, to further speed up the identification of the optimal extraction conditions, we took advantage of a Design of Experiment (DoE) approach, which is a statistical approach developed in the 1920s as an alternative to the one-factor-at-a-time approach. The strength of DoE is that it allows for maximizing information by minimizing the number of experiments required and thus minimizing environmental impact and costs (Cavalloro et al., 2024). According to greenness principles, in our DoE experimental plan only environmentally friendly solvents, such as a mixture of water and ethanol or pure ethanol, were used.

**FIGURE 5:**
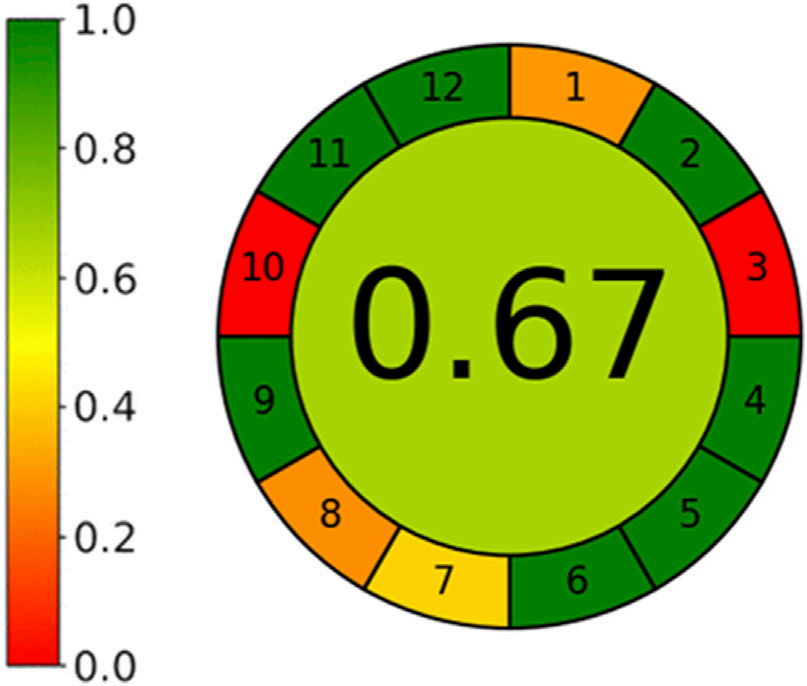
Greenness Assessment of the UHPLC method by Analytical GREennEss Calculator.

During the last decade, UHPLC proved to be significantly superior to HPLC protocols, guaranteeing shorter analysis time and smaller amount of mobile phase required. Furthermore, the UHPLC system requires a smaller injection volume and improves signal-to-noise ratio, thus enhancing sensitivity (Nahar et al., 2020). Following a green chemistry approach, we developed a UHPLC method suitable for the quantitation of both (*E)*-resveratrol and (*E*)-ε-viniferin in pruning wood extracts that can be considered a low impact procedure. Indeed, it required 24 min to draw the extract fingerprint, with a solvent consumption of 2.7 mL (water wasn't considered) per single run. Its greenness was confirmed by applying one of the most exploited open-source calculators in this field, namely “AGREE: analytical GREennEss Calculator 0.5 beta” (Yin et al., 2024; Pena-Pereira et al., 2020) that provides easily interpretable and informative results related to the environmental impact of analytical methods. AGREE starts from the 12 principles of green analytical chemistry (for details see reference Yin et al., 2024) and converts them into numerical values to assign a “greenness score” to analytical methods. Result of this software (0.67/1) demonstrated that our UHPLC method follows the 12 principles of Green Analytical Chemistry (Figure 5 see Reference Yin et al., 2024), with exception of Principle 3 (*In Situ* Measurements Should Be Performed), which is an intrinsic characteristic of chromatographic method that cannot be optimised, and Principle 10 (Reagents obtained from renewable source should be preferred), being bio solvents still poorly applied in chromatographic separations (Figure 5).

**FIGURE 6:**
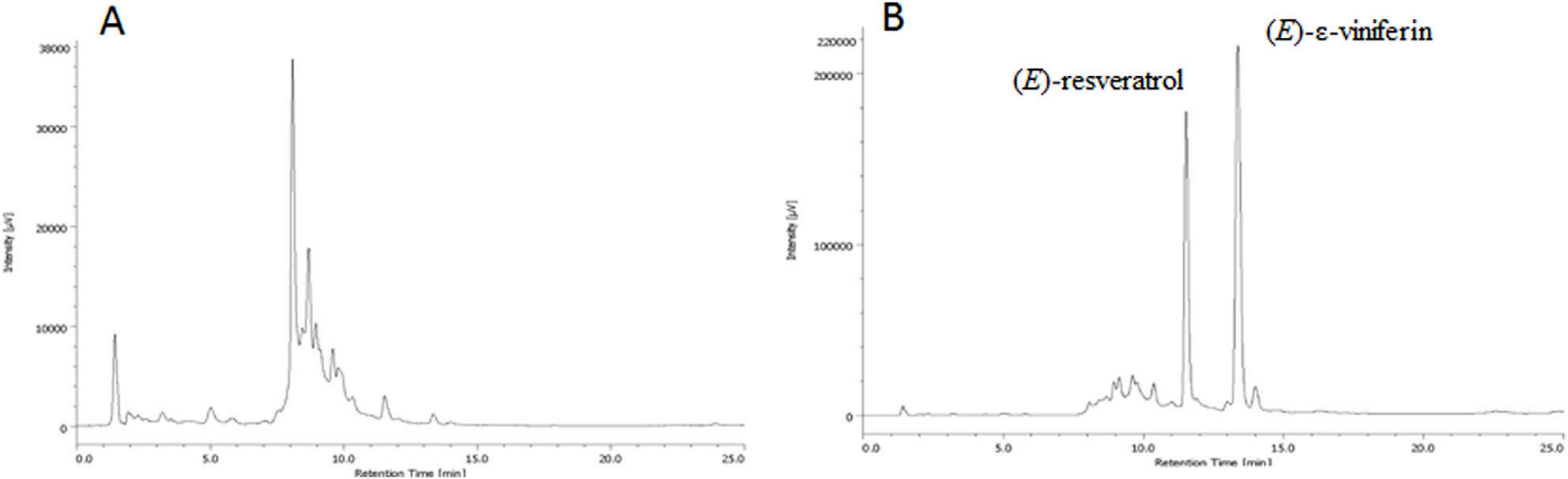
UV trace (λ: 306 nm) of the experiment #6 of Table 2 **(A)** and experiment # 7 of Table 2 **(B)**.

Concerning the extraction procedure, we exploited MASE due to its effectiveness in extracting secondary metabolites from natural matrices, as shown in numerous studies in recent years (Martino et al., 2019; Cavalloro et al., 2021). The use of MASE gives several advantages compared to conventional heat sources, as microwave energy is specifically targeted at the plant matrix, mainly improving extraction efficiency. Unlike conventional heat sources, microwaves heat the entire volume of the sample uniformly, taking advantage of two main phenomena: dipole rotation and ionic conduction, which act on the dipolar molecules and ions present in the sample, respectively. To speed up the identification of the optimal conditions for the extraction of the metabolites of interest, the experimental plan was set using a DoE approach, as summarised in Table 1. As previously discussed, the responses considered in the experimental plan were (*E*)-resveratrol and (*E*)-ε-viniferin extraction yields, *Y*_*1*_ and *Y*_*2*_, respectively, and the total extraction yield, *Y*_*3*_, while factors were water/ethanol ratio (*X*_*1*_), duration of a MASE cycle (*X*_*2*_) and temperature (*X*_*3*_ *).* The results of the output of the full factorial experiments (Table 2) highlighted no influence of the factors considered in *Y*_*3*_. Therefore, it is concluded that this response is robust to *X*_*1*_, *X*_*2*_, and *X*_*3*_ changes within the experimental domain. This result is explained by considering that although a high percentage of ethanol in water extracts phytocomplexes with a different fingerprint with respect to the ones obtained using a low percentage of ethanol in water (UHPLC-UV/DAD analysis), the total extraction yield of the crude extract remains approximately the same. In Figure 6 UHPLC-UV/DAD fingerprints of the extracts obtained using 80/20 (v/v) water/ethanol ratio (A, experiment #6 of Table 2) and 20/80 (v/v) water/ethanol ratio (B, experiment #7 of Table 2) are reported as an example.

Thus, we conclude that when *X*_1_, *X*_*2*_, and *X*_*3*_ are changed within the experimental domain investigated, the total extraction yield is described by the mean ± confidence limits at the 95% confidence level and it is robust to their variation. The yields of both (*E*)-resveratrol (*Y*_*1*_) and (*E*)-ε-viniferin (*Y*_*2*_) are influenced by the extraction solvent (*X*_*1*_), whereas they are robust to changes in *X_2_* (time of the microwave cycle) and *X*_*3*_ (Temperature). Such observation suggests that the extraction of (*E*)-resveratrol and (*E*)-ε-viniferin occurs more effectively when high percentages of ethanol in water are used (*X*_*1*_ = −1). Next, a second step of experiments was performed to further investigate the influence of ethanol in (*E*)-resveratrol and (*E*)-ε-viniferin yields. Particularly, three experiments using 100% ethanol were performed to test whether the extraction of both (*E*)-resveratrol and (*E*)-ε-viniferin could be improved. The yields of both metabolites increased, thus evidencing that the optimal extraction is achieved using 100% ethanol as extraction solvent, applying one cycle of microwave heating of 5 min at 80°C. This method proved to be easily scalable to tens-of-grams scale, as previously discussed.

As the last step of our work, once the best extraction parameters were identified, a fractionation protocol was exploited to obtain (*E*)-resveratrol and (*E*)-ε-viniferin in pure form, always keeping in mind eco-sustainable criteria. As previously reported, an important goal of the modern era is to reduce or eliminate waste generation in manufacturing processes. During the last decades, several metrics, like atom economy (AE), process mass intensity (PMI) or environmental impact factor (EF), have been developed for measuring the environmental impact of chemical processes. These metrics have gained interest in evaluating the greenness of processes in the field of petrochemicals, bulk/fine chemicals, or pharmaceuticals. However, only limited applications of these metrics have been reported in the literature. Focusing on EF, it is defined as “everything but the desired product” produced per kg of product, including solvent losses and chemicals used in work-up. It is calculated as follows:

$$EF=\frac{\Sigma m\;\left(input\;Matherials\;excl.\;water\right)-m\left(Product\right)}{m\left(Product\right)}$$

m = mass expressed in g.

The ideal EF value is zero, but it is generally considered acceptable in a range between 25 and 100 for pharmaceuticals or ≅ 0.1 for petrochemicals (Sheldon et al., 2022).

**FIGURE 7:**
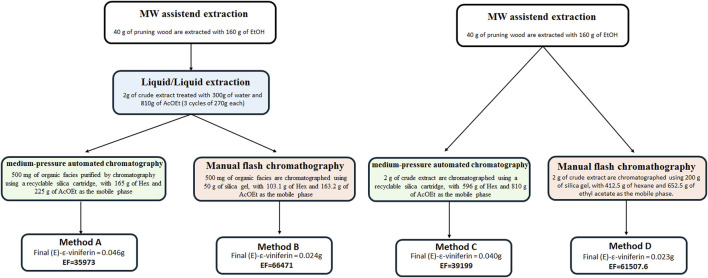
Comparison of methods A-D exploited for isolating (*E*)-resveratrol and (*E*)-ε-viniferin from *Vitis vinifera* pruning wood MASE extract (paragraph 2.7).

In our work, we exploited for the first time EF as a metric to compare different isolation procedures (methods A-D) of pure metabolites (E)-resveratrol and (E)-ε-viniferin (Figure 7). To the best of our knowledge, in the field of the extraction of active ingredients from natural sources, EF has already been applied only once for evaluating and comparing the extraction procedures of soybeans (Chea et al., 2025), but it has never been exploited for comparing processes focused on the isolation of pure metabolites. As can be noticed from the EF values related to method A-D (Figure 7), these are at least three magnitude orders higher if compared to those associated with pharmaceuticals. This can be explained by considering the significantly lower yield usually associated with the isolation of natural products if compared to synthetic chemicals. Furthermore, the EF values herein obtained are in line with the ones obtained in the previously cited publication without material recovery (Chea et al., 2025). Despite high, the EF values herein were easily obtained by applying the equation previously reported, allowing the quantitative comparisons among the different isolation processes. Particularly, we demonstrated that, despite being an additional fractionation step, the L/L extraction allows the reduction of the EF by minimizing the amount of the mobile phase required for the next chromatographic step (comparison between A vs. C or B vs. D, Figure 7). Furthermore, improvements have been achieved moving from flash manual to medium-pressure automated chromatography (comparison between A vs. B or C vs. D). This last change can be explained by considering both the possibility of recycling silica in the automated chromatography (influence directly correlated with the working scale) and the yield. Thus, the manual procedure caused a higher percentage of non-pure fractions, reducing the yield. At the end, based on EF calculations, we identified and proposed method A as a straightforward procedure to easily isolate both (*E*)-resveratrol and (*E*)-ε-viniferin from *Vitis vinifera* pruning wood MASE extract in good yields and high chemical purity (higher than 98%). Overall, the procedure involves microwave-assisted extraction of pruning wood, combined with liquid/liquid extraction, followed by automated medium-pressure liquid chromatography.

## 5 Conclusion

Global waste production is projected to increase significantly over the next four decades, presenting a major environmental challenge amid a rising global population. The urgent need for sustainable development and circular economy solutions has led our research to focus on vineyard pruning waste, an abundant viticultural byproduct rich in valuable secondary metabolites, particularly (*E*)-resveratrol and (*E*)-ε-viniferin, that are endowed with significant biological activity and industrial potential.

This study demonstrates an environmentally responsible process for extracting these high-value phytochemicals from what would otherwise be a discarded material. Our novel approach combines microwave-assisted solvent extraction (MASE) with optimized purification protocols specifically designed for isolating (*E*)-resveratrol and (*E*)-ε-viniferin. We systematically evaluated various purification techniques, including liquid-liquid extraction and chromatographic methods, both individually and in combination, to determine the most efficient protocol.

To support quantitative analysis throughout the process, we developed and validated a UHPLC-UV/DAD method for precise measurement of target compounds. Environmental sustainability was central to our methodology, therefore, we assessed the greenness of our analytical method using the AGREE calculator (Analytical GREEnness Calculator 0.5 beta) and evaluated the overall environmental impact of our extraction and isolation procedures using the environmental impact factor (EF). Both assessments yielded favourable results, confirming the eco-friendly nature of our approach.

This research provides a practical framework for converting agricultural waste into valuable bioactive compounds while minimizing environmental impact, representing a significant step toward more sustainable resource utilization in the agricultural sector. The residual plant material could be further used as a source of lignin or as a starting point to produce building materials.

## Data Availability

The original contributions presented in the study are included in the article/Supplementary Material, further inquiries can be directed to the corresponding author.
