# Supplementary material for: Vineyard pruning-wood waste valorisation: sustainable extraction of bioactive compounds
Source: Front Chem. 2025 Jun 5;13:1597833. doi: 10.3389/fchem.2025.1597833 (PMC12177893; doi:10.3389/fchem.2025.1597833)
Supplement: Supplementary file 1 [file DataSheet1.docx]

Supplementary Material

1. **Calibration curves of (*E*)-Resveratrol and (*E*)-ε-viniferin**

The calibration curves were built through ten points, each replicated three times

**
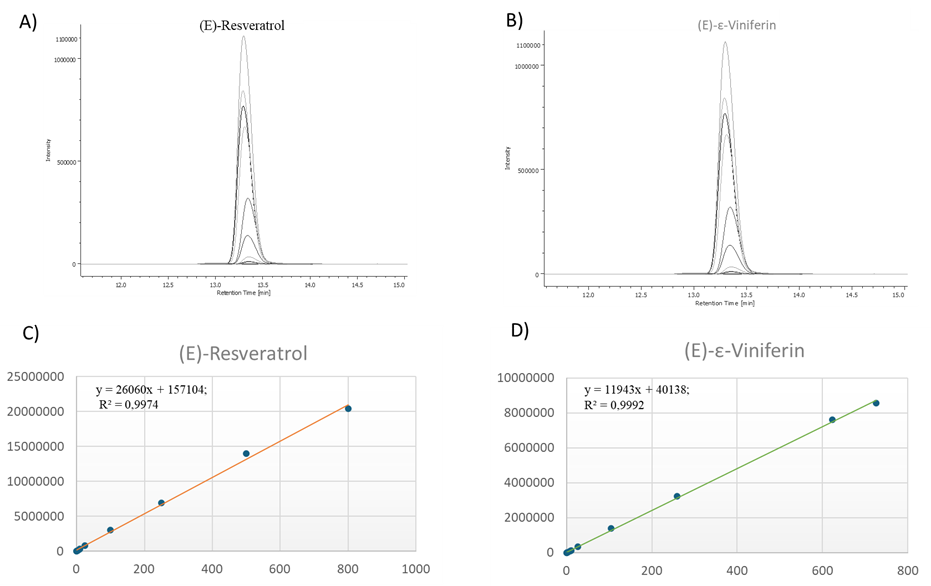
**

|  | **Range of linearity µg mL^-1^** | **DL µg mL^-1^** | **QL µg mL^-1^** |
| --- | --- | --- | --- |
| **(*E*)- Resveratrol** | **800-0.1** | **0.063** | **0.193** |
| **(*E*)- ε-Viniferin** | **726-0.1** | **0.070** | **0.212** |

**Supplementary Figure 1.** Range of calibration curves for (*E*)-Resveratrol and (*E*)-ε-viniferin
